# Deep Learning for Tumor Segmentation and Multiclass Classification in Breast Ultrasound Images Using Pretrained Models

**DOI:** 10.3390/s25247557

**Published:** 2025-12-12

**Authors:** K. E. ArunKumar, Matthew E. Wilson, Nathan E. Blake, Tylor J. Yost, Matthew Walker

**Affiliations:** 1 School of Agriculture and Food Systems, Davis College of Agriculture and Natural Resources, West Virginia University, Morgantown, WV 26506, USA; neb00001@mix.wvu.edu (N.E.B.); tjy0008@mix.wvu.edu (T.J.Y.); 2 West Virginia Agricultural and Forestry Experiment Station, Morgantown, WV 26506, USA; 3 Office of Statistics and Data Analytics, Davis College of Agriculture and Natural Resources, West Virginia University, Morgantown, WV 26506, USA; mwalke18@mix.wvu.edu

**Keywords:** breast cancer, ultrasound image analysis, artificial intelligence, pretrained models, transfer learning, U-Net, U-Net++, DeepLabV3, multiclass classification, Optuna optimization

## Abstract

Early detection of breast cancer commonly relies on imaging technologies such as ultrasound, mammography and MRI. Among these, breast ultrasound is widely used by radiologists to identify and assess lesions. In this study, we developed image segmentation techniques and multiclass classification artificial intelligence (AI) tools based on pretrained models to segment lesions and detect breast cancer. The proposed workflow includes both the development of segmentation models and development of a series of classification models to classify ultrasound images as normal, benign or malignant. The pretrained models were trained and evaluated on the Breast Ultrasound Images (BUSI) dataset, a publicly available collection of grayscale breast ultrasound images with corresponding expert-annotated masks. For segmentation, images and ground-truth masks were used to pretrained encoder (ResNet18, EfficientNet-B0 and MobileNetV2)–decoder (U-Net, U-Net++ and DeepLabV3) models, including the DeepLabV3 architecture integrated with a Frequency-Domain Feature Enhancement Module (FEM). The proposed FEM improves spatial and spectral feature representations using Discrete Fourier Transform (DFT), GroupNorm, dropout regularization and adaptive fusion. For classification, each image was assigned a label (normal, benign or malignant). Optuna, an open-source software framework, was used for hyperparameter optimization and for the testing of various pretrained models to determine the best encoder–decoder segmentation architecture. Five different pretrained models (ResNet18, DenseNet121, InceptionV3, MobielNetV3 and GoogleNet) were optimized for multiclass classification. DeepLabV3 outperformed other segmentation architectures, with consistent performance across training, validation and test images, with Dice Similarity Coefficient (DSC, a metric describing the overlap between predicted and true lesion regions) values of 0.87, 0.80 and 0.83 on training, validation and test sets, respectively. ResNet18:DeepLabV3 achieved an Intersection over Union (IoU) score of 0.78 during training, while ResNet18:U-Net++ achieved the best Dice coefficient (0.83) and IoU (0.71) and area under the curve (AUC, 0.91) scores on the test (unseen) dataset when compared to other models. However, the proposed Resnet18: FrequencyAwareDeepLabV3 (FADeepLabV3) achieved a DSC of 0.85 and an IoU of 0.72 on the test dataset, demonstrating improvements over standard DeepLabV3. Notably, the frequency-domain enhancement substantially improved the AUC from 0.90 to 0.98, indicating enhanced prediction confidence and clinical reliability. For classification, ResNet18 produced an F1 score—a measure combining precision and recall—of 0.95 and an accuracy of 0.90 on the training dataset, while InceptionV3 performed best on the test dataset, with an F1 score of 0.75 and accuracy of 0.83. We demonstrate a comprehensive approach to automate the segmentation and multiclass classification of breast cancer ultrasound images into benign, malignant or normal transfer learning models on an imbalanced ultrasound image dataset.

## 1. Introduction

Breast cancer is the leading cause of cancer-related deaths among women. A 2024 study showed that around 1 in 8 women (13%) will be diagnosed with breast cancer and 1 in 43 (2.3%) will die from the breast cancer [1]. This type of cancer develops in the breast tissue and can form tumors that are detectable using various imaging sensors. Among the various sensing technologies used in clinical diagnostics, ultrasound sensors play a crucial role due to their ability to capture real-time, high-resolution images from soft tissue without exposing patients to ionizing radiation. Unlike X-ray-based sensors, ultrasound transducers are safer for repeated use, more cost-effective and widely accessible in clinical settings [2,3]. As the senor transmits acoustics waves and measures the reflected signals, it produces ultrasound images that reveal variations in tissue density and potential abnormalities.

Sensor-acquired breast ultrasound images are categorized into three classes: normal images, benign tumor images and malignant tumor images. Accurate classification of these sensor outputs is essential for effective diagnosis. However, traditional assessment of breast ultrasound sensor data presents challenges such as the fact that subtle signs of malignancy often resemble benign patterns, especially when lesions lack clear boundaries in the sensor-generated images [4]. Such overlap can lead to false negatives, where malignant tumors are mistaken for benign tumors, and false positives, where normal or benign tissue is incorrectly judged as malignant. Medical image segmentation, derived from sensor outputs, is equally challenging. Image processing techniques are essential for the delineation of lesions, enabling quantification of tumor size, shape and volume.

Segmentation of ultrasound sensor data helps radiologists concentrate on the region of interest by isolating relevant patterns from surrounding healthy tissue [5]. However, manual image segmentation and classification of ultrasound images are time-consuming and prone to variability due to differences in radiologists’ experience and interpretation styles [6]. Such variability increases diagnostic uncertainty and the risk of misdiagnosis. Therefore, the development of AI tools is critical to enhance the reliability and efficiency of the breast cancer diagnosis process, particularly image segmentation and classification of tumors. By training deep learning models on extensive and diverse ultrasound sensor image datasets, these systems can learn to detect subtle edges and intricate patterns that may not be visible to the human eye. Hence, the present work aims to develop automated breast cancer detection tools that perform both segmentation and classification of breast ultrasound images using transfer learning with pretrained model weights.

Recent developments in Convolutional Neural Networks (CNNs) have provided outstanding solutions for medical image analysis [7,8,9]. Fully convolutional networks (FCNs) and U-Shaped convolutional neural networks (U-Nets) are cutting-edge deep learning models designed to handle complex input data. These models use deep neural nets to process data by hierarchically extracting features via hidden layers and iteratively training the networks [10]. Soulami et al. developed a U-Net model using mammogram datasets, achieving remarkable metrics—an IoU of 90.50% and an AUC of 99%. However, it was limited by training from scratch, relying on a single architecture and binary classification and focusing solely on mammogram images, which are less effective for dense breast tissue and involve radiation exposure [11,12]. Additionally, a fuzzy logic network in combination with eight CNN-based pretrained models was trained to perform semantic segmentation on the BUSI dataset, a publicly available 2019 collection of expert-labeled breast ultrasound images grouped into normal, benign and malignant categories. However, the authors did not discuss hyperparameter tuning or how class imbalance in the BUSI dataset was addressed [13].

In another study, a hybrid CNN–transformer network was developed for breast cancer ultrasound image segmentation by using transformer encoder blocks in the encoder part of the CNN–transformer network to learn the global contextual information, then combined with a CNNs to extract features. In the decoder part, they used a spatial cross-attention module to reduce the semantic discrepancy with the encoder. These models achieved high accuracy; however, a transformer-based architecture requires extensive computational resources, large-scale pretraining and a complex attention mechanism [14]. Guo et al. developed U-Net for semantic segmentation of breast cancer ultrasound images and reported average Dice and IoU coefficients of 90.5% and 82.7%, respectively [15]. In 2024, Nastase et al. developed and tested two segmentation architectures for delineating lesions using the BUSI dataset. They reported that DeepLabV3 outperformed the baseline U-Net model on segmentation tasks, with average Dice scores of 93% and 90% in predicting ground-truth masks (tumor or background); however, this work did not address the class imbalance present in the BUSI dataset, which indicates limited generalizability and that biases likely exist in the model. Moreover, this study excluded benign images in the investigation, making the system clinically less relevant [16]. Recent studies have developed advanced segmentation models [17,18]. Zhao et al. recently introduced a deep learning framework based on the Short-Term Dense Connection (STDC) architecture for Mura defect detection in Micro-OLED displays. Their model incorporates Depth-Wise Separable Atrous Spatial Pyramid Pooling (DW-ASPP) and a Coordinate Feature Fusion Module (CFFM) to enhance multi-scale feature extraction, spatial–channel fusion and overall efficiency [17]. However, this model has not been tested for its ability to segment ultrasound or radiology datasets. Similarly, frequency-domain representations have gained attention in deep learning. Qin et al. [19] introduced a frequency channel attention network (FcaNet), while Rao et al. [20] proposed global filtering using Fast Fourier Transform (FFT) for vision transformers (GFNet). However, these methods focus on general computer vision tasks and do not address the specific challenges of medical ultrasound imaging, where speckle noise and low contrast require specialized frequency handling.

In a study of the performance of the FCN, U-Net, DenseNet121 and PSPNet models in terms of IoU, accuracy, precision and F1 score (the harmonic mean of precision and recall, reflecting the model’s ability to correctly identify positive cases while minimizing false positives and false negatives), their model achieved 95% prediction accuracy in terms of image segmentation of a breast cancer histology dataset [21]. Similarly, transfer learning techniques were used to perform binary and multiclass classification of breast cancer ultrasound images [22]. A comparison of ResNet50, ResNeXt50 and VGG16 models in classifying breast cancer ultrasound images reported that ResNetXt50 achieved an accuracy of 85.83%, ResNet50 achieved an accuracy of 85.4% and VGG16 achieved the lowest accuracy of 81.11% [22]; however, this study did not include an external validation set to test the models’ generalizability. In another study, a model inspired by GoogleNet and residual blocks with learnable activation functions was developed to conduct image classification [23]. The authors reported that their model showed an accuracy of 93% on breast cancer ultrasound images, with F1 scores ranging from 0.88 to 0.93 across different model variants, with corresponding loss value ranging between 0.21 and 0.3 [23].

Various approaches have been proposed in the literature to address the problem of breast cancer segmentation and classification using the BUSI dataset and image classification in general [17], but there are fewer comprehensive reports that include both the development of segmentation and classification models based on transfer learning for breast cancer image segmentation and multiclass classification. Hatamizadeh et al., introduced UNET Transformers (UNETR) network [24] which was optimized by Said et al., to conduct image segmentation and classification on a lung cancer image dataset. They reported that their model achieved accuracies of 97.83% for image segmentation and 98.77% for classification [25].

The goal of this study is to develop automated breast cancer detection tools that perform both segmentation and classification of breast ultrasound images using transfer learning with pretrained model weights. This work evaluates the performance of U-Net, U-Net++ and DeepLabV3 segmentation models using an encoder–decoder design with pretrained encoders (ResNet18, EfficientNet-B0 and MobileNetV2). In addition, we develop a multiclass classification model for classification of BUSI images as malignant, benign or normal. To this end, we developed and optimized pretrained models including ResNet18, InceptionV3, DenseNet121, MobileNetV3 and GoogleNet. The major contribution of this work includes the introduction of a novel FADeepLabV3 model, which incorporates a lightweight FEM into the DeepLabV3 architecture. Unlike prior frequency-oriented models [26,27], this novel FEM approach performs localized frequency decomposition within each feature map, separating low-, mid- and high-frequency components using FFT-based analysis and applying band-specific learnable refinements before residual fusion. This hybrid spatial–frequency design strengthens boundary localization while maintaining the computational efficiency and spatial modeling strengths of DeepLabV3. Although this study evaluates FADeepLabV3 on breast ultrasound images, where speckle noise, low contrast and poorly defined lesion boundaries make frequency-band modeling particularly beneficial, the proposed FEM is architecture-agnostic and can be integrated into any encoder–decoder network. Beyond this architectural innovation, all segmentation and classification models were optimized through systematic hyperparameter tuning using Optuna.

## 2. Materials and Methods

### 2.1. Dataset

**Figure 1:**
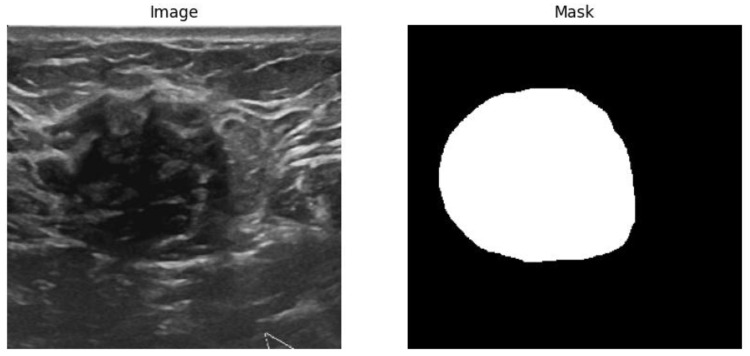
Sample image and mask pair data for image segmentation task.

**Figure 2:**
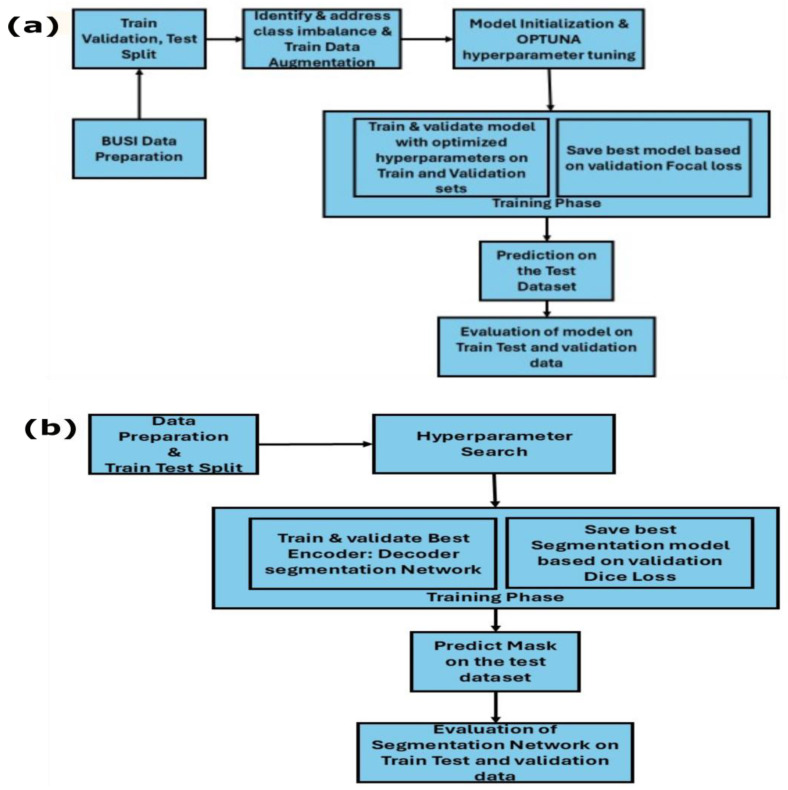
(**a**) Breast cancer segmentation using encoder–decoder architecture. (**b**) Multiclass classification of breast cancer images.

The BUSI dataset [28] was utilized for both segmentation and classification tasks in this study. Figure 1 shows sample input data of the BUSI dataset. Image segmentation and classification of the BUSI dataset remained a challenging task due to its poor image quality [29]. This dataset contains 780 images, categorized into three classes: normal (132), benign (436) and malignant (210) as shown in Table 1. We developed workflows for both segmentation (Figure 2a) and classification (Figure 2b). The first step of the development process is data preparation; in this step, the dataset was divided into training, validation and test sets using an 80:10:10 split, and proportional representation of each class was ensured across the sets. In this study, training and validation datasets were used in the training phase of the models, and the test dataset was only used in the inference phase to evaluate model performance and generalization on unseen data. Table 2 shows the distribution of the images specific to each label. From Table 2, it is evident that the dataset is imbalanced, containing a greater number of instances with the “benign” label compared to “normal” or “malignant” samples.

### 2.2. Segmentation Models

The U-Net, U-Net++ and DeepLabV3 segmentation models were trained and tested to predict the masks of the BUSI dataset to segment the lesions of the images.

#### 2.2.1. U-Net

**Figure 3:**
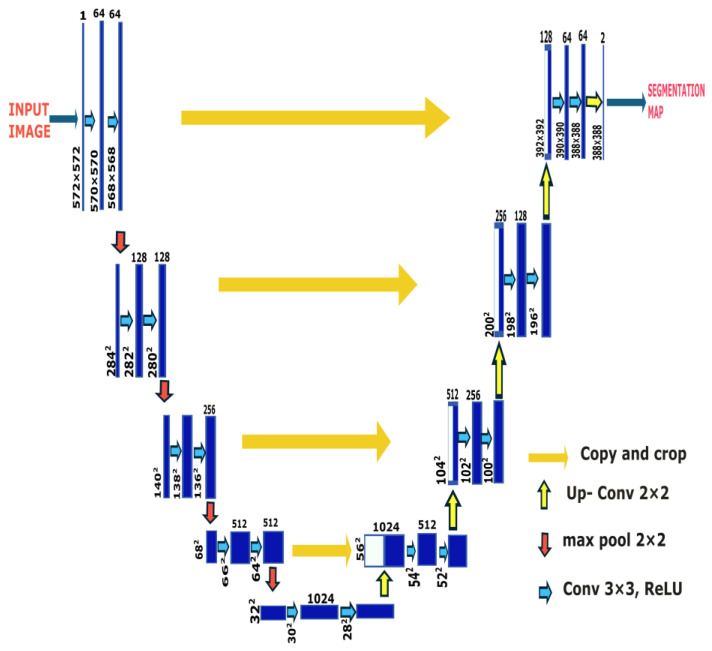
U-Net architecture for medical image segmentation. The encoder progressively downsamples the input image through convolutional and max pooling layers, while the decoder upsamples feature maps through transposed convolutions and skip connections, producing a pixel-wise segmentation map [30].

U-Net is a CNN model architecture (Figure 3) [30] originally designed to work well on the smaller medical imaging datasets commonly used in biomedical engineering [31]. Based on a fully convolutional network (FCN), the U-Net architecture is expanded to include encoder–decoder paths that are designed to contract and expand the features of the input images. The encoder captures the context of the input image by gradually reducing the spatial dimensions while increasing the feature depth through a series of 3 × 3 convolutions, followed by Rectified Linear Units (ReLUs) and 2 × 2 max pooling operations. These operations are mathematically represented by Equations (1) and (2). Max pooling reduces the spatial resolution of feature maps from 572 × 572 to 28 × 28 while retaining the most salient features by increasing the number of feature channels from 64 to 1024.

(1) $$Y=\sigma \left(X*W+b\right)=max(0,X*W+b)$$

(2) $$Y_{i,j}=max\{X_{m,n}:(m,n)\in R_{i,j}\}$$

where σ is the rectified linear Unit (ReLU) activation function, *X* is the input, *W* is a learnable filter with a bias of *b* to generate feature map *Y* and $R_{i,j}$ is the receptive field (pooling window) corresponding to output position (i,j). The max pooling operations are applied at each encoder level, gradually capturing the high-level features.

The decoder architecture reconstructs the segmentation map by upsampling the feature maps via 2 × 2 transposed convolutions, halving the number of feature channels at each level to match the original input resolution. Skip connections between encoder and decoder layers concatenate feature maps from corresponding encoder layers to the decoder layer, preserving spatial information lost during downsampling. Feature maps ($X_{enc}^{\left(l\right)}$) are concatenated with corresponding feature maps of the decoder ($X_{dec}^{\left(l\right)}$), improving spatial accuracy in segmentation, as shown in Equation (3). The final 1 × 1 convolution maps the 64-channel feature representation to the designed number of segmentation classes, producing an output segmentation map.

(3) $$X_{dec}^{\left(l+1\right)}=f(concat({X_{enc}^{\left(l\right)},\;\;Up(X}_{dec}^{\left(l\right)})))$$

where concat represents concatenation along the feature dimension, *Up*() denotes the upsampling operation and f represent the subsequent convolutional layers applied at each decoder stage. In the final layer, 1 × 1 segmentation is applied to map the output to the desired number of classes, producing a segmentation map by classifying each pixel, as shown in Equation (4).

(4) $$Z=softmax(X_{dec}^{\left(L\right)}.W_{final}+b)$$

where $X_{dec}^{\left(L\right)}$ is the output of the last decoder layer and $W_{final}$ is the 1 × 1 filter that maps to channels, with each channel representing one class probability, normalized by the SoftMax function. An in-depth mathematical explanation of the U-Net architecture can be obtained in recently published research work [32].

#### 2.2.2. U-Net++

**Figure 4:**
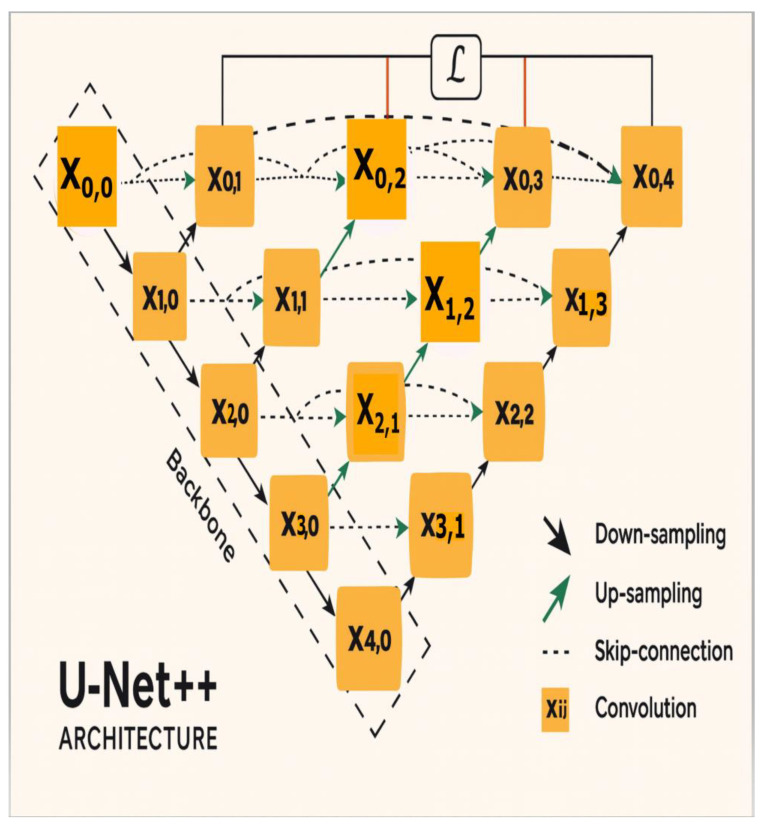
U-Net++ architecture, demonstrating dense skip connections across multiple scales. The network features a nested encoder–decoder structure with sequential convolutional layers ($X^{i,j}$) connected through downsampling, upsampling and skip-connection pathways, enabling multi-scale feature extraction and fusion for improved segmentation [33].

U-Net++ (Figure 4) [33] is an extension of the standard U-Net architecture that introduces two key structural improvements: nested skip pathways and deep supervision [34,35]. Unlike the direct skip connections in U-Net, encoder–decoder pathways of U-Net++ are interconnected through nested, dense convolutional blocks [35]. Each decoder node receives concatenated feature maps from all preceding encoder levels, enabling hierarchical feature aggregation across multiple semantic scales. This dense connectivity pattern progressively reduces the semantic gap between encoder and decoder representations, enabling effective feature fusion at each resolution level. Feature map $X_{i,j}$ connects encoder level i to decoder level j. The mathematical representation of the convolutional blocks is given by Equation (5).

(5) $$X_{ij}=f(concat(X_{i,\;j-1},X_{i-1,j}X_{i-1,\;j+1},\;\ldots ))$$

where $X_{i,j}^{\left(l\right)}$ is the feature map at encoder level i and decoder level j after l convolutional layers, concat() is concatenation across the feature dimensions and f represents convolutional layers applied to the concatenated feature map. Deep supervision [34] enables the model to operate in “accurate” mode or “fast” mode. In accurate mode, the outputs from all segmentation branches are averaged, whereas in “fast” mode, the final segmentation map is selected from only one of the segmentation branches. The choice between the two modes results in model pruning and speed gain. This dual-mode capability allows for model pruning during inference, providing a controllable trade-off between segmentation accuracy and computational efficiency during deployment.

#### 2.2.3. DeepLabV3

**Figure 5:**
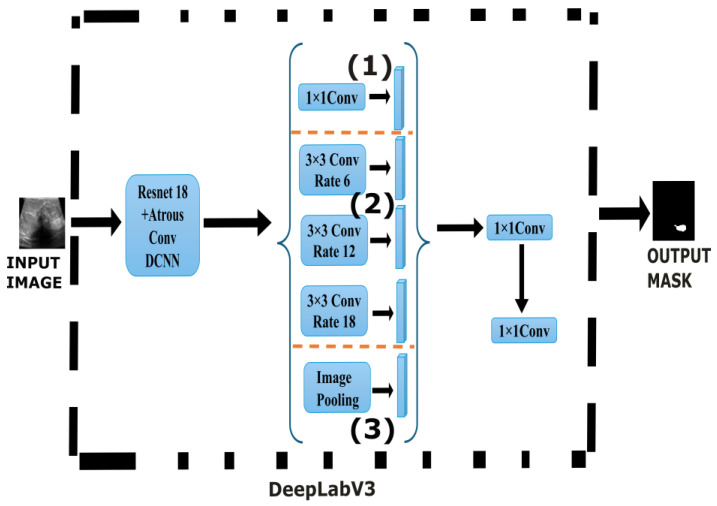
DeepLabV3 architecture. The core ASPP module captures multi-scale context via parallel branches: **(1)** convolution, **(2)** three atrous convolutions with increasing dilation rates and **(3)** a global average pooling branch.

DeepLabV3 (Figure 5) [36] was proposed by the Google Brain team [37] to address spatial resolution loss inherent in deep CNNs caused by consecutive pooling and striding operations. It uses the concept of ASPP [38] to control the receptive fields, thereby capturing the wider context of the images without reducing the image resolution. DeepLabV3 addresses this issue by using cascading atrous convolutions with varying rates to capture multiscale contextual information. The ASPP module consists of parallel convolutions with multiple rates (6,12,18), a 1 × 1 convolution for capturing fine-grained channel information and global average pooling followed by 1 × 1 convolution and upsampling to encode image-level features that provide global context. By applying ASPP convolutions at different rates, the network can effectively capture objects and context at multiple scales without computational burden. A detailed mathematical explanation and a description of the research methodology of DeepLabV3 are provided in [37].

#### 2.2.4. FADeepLabV3

**Figure 6:**
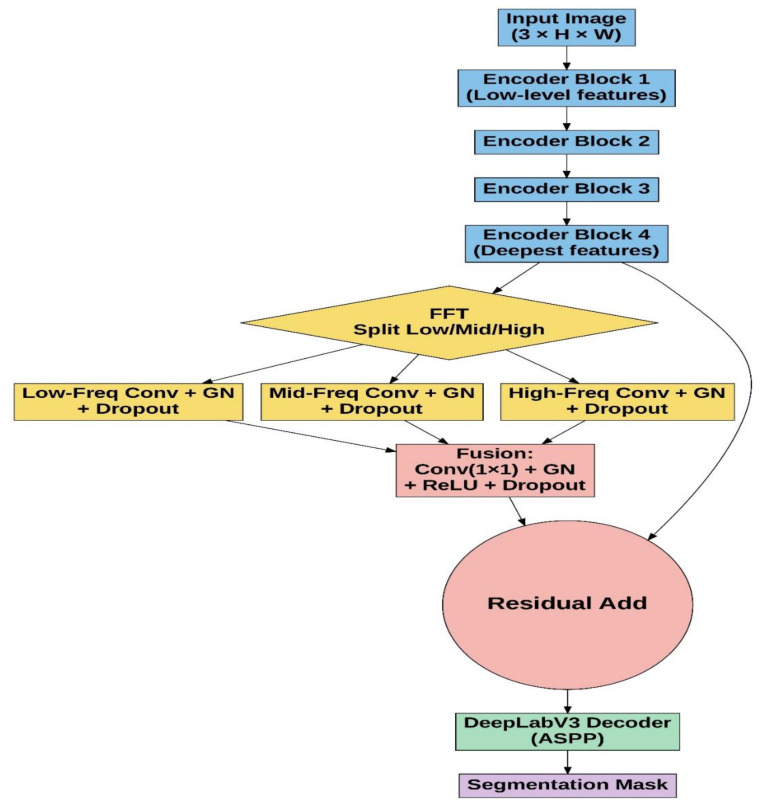
Architecture of Frequency-Aware DeepLabV3 for semantic segmentation. The network consists of a Resnet encoder (blue), frequency enhancement module (yellow), feature fusion module with residual connection (pink), DeepLabV3 decoder with ASPP (green) and predicted mask (purple). The deepest encoder features undergo FFT-based decomposition into low-, mid- and high-frequency components, which are processed independently through convolutional layers with group normalization and dropout before fusion. A residual connection preserves spatial information while incorporating frequency-domain enhancements.

The proposed FADeepLabV3 model builds upon the standard DeepLabV3 architecture by integrating an FEM that processes deep encoder features in the frequency domain before the decoder path. This module enhances feature representation by decomposing features into multiple frequency bands. The model learns global lesion morphology through low-frequency components and local boundary structures via high-frequency components. FADeepLabV3 was strategically applied to the deepest fourth block of the Resenet18 encoder path, where receptive fields are largest and semantic information is most abstract, providing an optimal foundation for frequency decomposition and processing. The architecture of the proposed FEM is illustrated in Figure 6. Given the deepest encoder feature map (Equation (6)), the FEM enhances lesion-related structure through frequency-domain decomposition, band-wise refinement and residual fusion before passing features to the DeepLabV3 decoder.

(6) $${X\in R}^{B\times C\times H\times W}$$

In Equation (6), the spatial feature tensor is transformed into the frequency domain using the 2D real-valued FFT (rFFT2) [39], which is mathematically represented by Equation (7), producing a compact spectrum (Equation (8)).

(7) $$\hat{x}=rFFT2(X)$$

(8) $$\hat{x}\;\in C^{B\times C\times H\times W_{freq}}$$

where $W_{freq}$ = [*W*/2] + 1, which denotes compressed frequency width returned by *rFFT2*. Then, the spectrum is partitioned into three non-overlapping spectral regions using simple binary masks ($M_{\mathrm{low}},M_{\mathrm{mid}},M_{\mathrm{high}}$). By multiplying the spectrum by each mask, the module extracts low-frequency bands for global structure and coarse lesion shapes, mid-frequency bands for intermediate structural transitions and high-frequency bands for sharp boundaries and subtle edge details. This is mathematically represented by Equation (9).

(9) $${\hat{x}\;}_{k}=\hat{x}\;⊙M_{k},\;K\in \{low,\;mid,\;high\}$$

These three parallel paths correspond to Low-Freq, Mid-Freq and High-Freq operations. Each masked component is returned to the spatial domain, yielding three band-limited feature maps aligned with the original spatial resolution using Inverse Real-valued 2D Fast Fourier Transform (irFFT2), preserving the global structure, with mid frequencies reflecting anatomical edges and high frequences enhancing the lesion boundaries.

(10) $$X_{k}=\mathrm{irFFT}2({\hat{X}}_{k})\in \{low,\;mid,\;high\}$$

Each spatial band undergoes lightweight refinement and is reduced individually, as shown in Equation (11), followed by channel-wise bottleneck convolution and fusion (Equation (12)) by passing through a two-layer fusion module that learns the cross-frequency interactions and restores original channel dimensionality; then, the fused representation is integrated with the original encoder feature via residual addition (13), which ensures that the module refines rather than replaces semantic features. Then, the FEM is inserted at the final encoder layer of DeepLabV3, which is mathematically represented by Equation (14). The ASPP decoder receives the modified feature map to generate the final segmentation map.

(11) $$F_{k}=\mathrm{Dropout}(\mathrm{GN}({\mathrm{Conv}}_{1\times 1}(X_{k})))$$

(12) $$F={Conv}_{1\times 1}([F_{low}∥F_{mid}∥F_{high}])$$

(13) $$Y=X+Dropout(F_{fused})$$

(14) $$Y=FEM(X_{deepest})$$

### 2.3. Classification Models

#### 2.3.1. ResNet18

In 2015, He et al. introduced deep residual networks (ResNet) for image recognition [40]. ResNet18 consists of 18 convolutional layers, with an input layer with an image size of 3 × 244 × 244. ResNet18 contains four blocks of neural networks, each block containing two basic locks of two convolutional layers, with the last layer being a fully connected layer, for a total of 18 layers. This network solves the vanishing gradient problem through skip connections, which add the input of the block to its output. ResNet learns identity mapping using skip connections and is suitable for various image segmentation and classification tasks.

#### 2.3.2. GoogleNet

In 2015, GoogleNet, a CNN architecture also known as InceptionV1, was proposed by Szegedy C et al. [41] based on the Network in Network structure proposed by Lin M in 2013 [42]. The inception architecture increases the network width by using multi-input layers and multiple convolution kernels of different sizes to capture a wide range of details from input images. GoogleNet has 22 network layers, but only 1/36th of the parameters of the VGG [43].

#### 2.3.3. InceptionV3

InceptionV3, developed by the Google Brain team, achieved a top-five error rate of 3.46% on the ImageNet dataset. In 2016, Google released the latest version of the inception model, containing 48 layers and introducing factorized convolutions, where a 5 × 5 kernel is replaced with two 3 × 3 convolutions to maintain performance while reducing computational cost. The input layer of InceptionV3 receives images with dimensions of 3 × 299 × 299, where 3 corresponds to the RGB color channel and 299 × 299 represents the spatial resolution (height and width) of the input image.

InceptionV3 consists of three components: an initial convolutional block, multiple inception modules and a final classifier [44]. The initial convolutional block contains alternative convolutional and max pooling layers to extract features from the input image. The inception modules perform multiscale convolutions in parallel using kernels of different sizes (1 × 1, 3 × 3 and 5 × 5), followed by concatenation to capture diverse features [45]. The final classifier includes fully connected layers, with a SoftMax layer at the end to output class probabilities for the target classes.

#### 2.3.4. MobileNetV3

MobileNetV3 [46] is designed for efficient image classification—specifically, to run on mobile and edge devices. This model was released in 2019 by the Google Brain team. The input layer of MobileNetV3 accepts images with dimensions of 3 × 244 × 244. It consists of a convolutional block, multiple MobileNetV3 modules and a final classifier. The primary features are extracted using the initial convolutional block with batch normalization and a ReLU activation function. The core of the architecture includes squeeze-and-excitation (SE) modules embedded in the bottleneck and inverted residual blocks with depth-wise separable convolutions.

#### 2.3.5. DenseNet121

DenseNet121 introduces dense connectivity between layers; each layer is directly connected to every subsequent layer within a dense block, creating a densely connected network structure. Unlike CNNs, where the information passes subsequently from one layer to the next, DenseNet121 contains direct pathways from each layer to all subsequent layers through feature map concatenation [47].

The network is composed of three main components: a convolutional block, multiple dense blocks connected by transition layers and a final classifier. A transition layer positioned between the dense blocks consists of batch normalization followed by a 1 × 1 convolution and a 2 × 2 average pooling operation, which collectively reduce the number and spatial dimensions of feature maps to maintain computational efficiency. In the dense block of *L* layers, each layer *(l)* receives the feature maps of all preceding layers [0, …, l − 1] as input, leading to L(L+1)/2 connections. Each layer adds its own k feature maps to the network’s collective knowledge, where k is the growth-rate hyperparameter. This means the Lth layer has $K_{0}+K(l-1)$ input features, where $K_{0}$ is the initial number of features. These dense connections allow for feature reuse, strengthen gradient flow and solve the vanishing gradient problem by providing multiple paths for information propagation.

### 2.4. Evaluation Metrics

The performance of the proposed models was tested using several standard evaluation metrics that are widely used in image segmentation and classification tasks. These metrics provide a comprehensive account of the model’s effectiveness in terms of accuracy, precision and reliability.

#### 2.4.1. Intersection over Union (IoU)

The intersection over union, also known as the Jaccard Index, is used to evaluate segmentation quality. It measures the overlap between the predicted segmentation mask and the ground-truth mask. The IoU penalizes both over-segmentation and under-segmentation, providing a balanced assessment of the segmentation accuracy. The IoU is mathematically expressed as follows:

(15) $$IoU=\frac{\left|A⋂B\right|}{\left|A⋃B\right|}$$

where *A* represents the predicted segmentation mask and *B* is the ground-truth mask.

#### 2.4.2. Confusion Matrix

The confusion matrix provides a detailed breakdown of the model’s performance across different classes. It provides a tabular summary of the predicted class labels with the true class labels, offering detailed insights into the model’s performance across all classes. For a classification task with *n* class labels, the confusion matrix is an n×n table, where rows represent the actual classes and columns represent the predicted classes. The table contains True Positives (TPs), cases correctly predicted as positive cases; False Positives (FPs), classes where the model incorrectly predicted the positive class; False Negatives (FNs), classes where the model incorrectly predicted the negative class; and True Negatives (TNs), cases correctly predicted as the negative class.

#### 2.4.3. Pixel Accuracy

Pixel accuracy represents the proportion of correctly classified pixels across all classes. It is calculated as shown in Equation (16). In this study, we consider binary-class image segmentation (background vs. mask); hence *k* = 1.

(16) $$PA=\frac{\sum_{i=0}^{k}p_{ii}}{\sum_{i=0}^{k}\sum_{i=0}^{k}p_{ij}}$$

#### 2.4.4. AUC

The area under the receiver operating characteristic (ROC) curve, commonly known as the AUC, evaluates the model’s ability to distinguish between classes across various classification thresholds. The AUC score ranges from 0 to 1 where, AUC = 1 represents perfect classification, AUC = 0.5 indicates random classification and AUC < 0.5 suggests worse-than-random classification.

#### 2.4.5. Dice and Focal Loss

Dice Loss (DL) is a loss function that measures the overlap between predicted segmentation masks and the ground-truth masks. DL is derived from the Dice Similarity Coefficient (DSC), which is used to find the similarity between two sets. DL is particularly useful when dealing with imbalanced datasets because it emphasizes regions of interest to improve the accuracy of segmentation models by prioritizing overlap. The DSC is defined as shown in Equation (17).

(17) $$DSC=\frac{2|X\cap Y|}{\left|X\right|+|Y|}$$

where X is the set of predicted pixels and Y is the set of actual pixels. |X∩Y| is the number of pixels common to both *X* and *Y.* The DSC can also be mathematically represented in terms of individual pixels and ground-truth pixels, as shown in Equation (18).

(18) $$Dice\;Coefficient=\frac{2\sum_{i=1}^{N}p_{i}g_{i}}{\sum_{I=1}^{N}p_{i}+\sum_{i=1}^{N}g_{i}}$$

where $p_{i}$ is the predicted probability for the $i^{-th}$ pixel, $g_{i}$ is the ground-truth label for the $i^{-th}$ pixel and N is the total number of pixels. The Dice loss is then calculated using the Dice coefficient as shown in the Equation (19).

(19) DL=1−DSC

Similarly, for the classification task, focal loss was used to optimize the models during the training process. Focal loss was introduced in 2017 and is designed to address class imbalance by focusing on hard-to-classify instances [48]. It is a modification of the cross-entropy loss, adding a modulating term ($1-p_{c}{)}^{\gamma }$, reducing the loss contribution of well-classified instances and allowing the model to focus on hard cases. For correctly classified samples, $p_{c}$ is close to 1 and (*1* − $p_{c}$) approaches 0, reducing the contribution of such samples to the loss. When γ *=* 0, the term ($1-p_{c}{)}^{\gamma }$ is equal to 1, which simplifies to standard cross-entropy. For a three-class classification task, the focal loss that is minimized during the training process is mathematically defined as shown in Equation (20).

(20) $$FL=-\sum_{c=1}^{3}\alpha_{c}\times (1-p_{c}{)}^{\gamma }\times y_{c}\times log(p_{c})$$

where *c* represents classes 1, 2 and 3 and $\alpha_{c}$ is the class-specific weight factor for class *c*. The value of *c* lies between 0 and 1, giving more weight to under-represented classes in the imbalanced dataset. $y_{c}$ is a variable that ensures that *FL* is focused on the true class during training. $y_{c}$ = 1 if class c is the true class for the sample and 0 otherwise. To evaluate the performance of the models on the training, test and validation datasets, precision, recall, F1 score and their macro average were calculated from the confusion matrix results. Macro averaging is a technique used to address datasets that contain imbalanced classes, treating each class equally and not weighting larger classes more than smaller classes. $log(p_{c})$ is a penalty term that penalizes incorrect predictions based on the model’s confidence level, while $p_{c}$ is model’s predicted probability for class c for a given sample. For the true class $p_{c}$ is high; the further $p_{c}$ is from 1, the higher the penalty imposed by the loss function.

#### 2.4.6. Precision and Recall

Precision measures the proportion of predicted positive instances that are correctly classified. It is defined as the ratio of true positives to the total number of positive predictions (both true and false), as shown in Equation (21). Recall, also known as sensitivity, measures the ability of the model to correctly identify all relevant positive instances (ground truth). It is mathematically represented by Equation (21).

(21) $$Precision=\frac{TP}{TP+FP}$$

(22) $$Recall=\frac{TP}{TP+FN}$$

where *TP* is the true-positive count (correctly predicted positive instances), *FP* is the false-positive count (incorrectly predicted positive instances) and *FN* is the false-negative count (instances that were positive but predicted as negative)

#### 2.4.7. F1 Score

F1 score is the harmonic mean of precision and recall. It is commonly used on imbalanced datasets, since the harmonic mean emphasizes the lower value between precision and recall. Thus, the F1 score will be lower if either precision or recall is low, reflecting the performance of the model more accurately in cases where there is an imbalance between the two metrics. The F1 score is mathematically represented by Equation (23).

(23) $$F1=2\times \frac{Precision\times Recall}{Precision+Recall}$$

## 3. Approach

In this study, image segmentation architectures were trained on images, with the corresponding segmentation masks as ground-truth labels. In the classification task, the models were trained solely on the images, with the target labels being normal, benign and malignant. Since deep learning models require a large amount of data to achieve ideal performance and the BUSI dataset is relatively small, data augmentation techniques were applied to increase the effective size of the training set.

**Figure 7:**
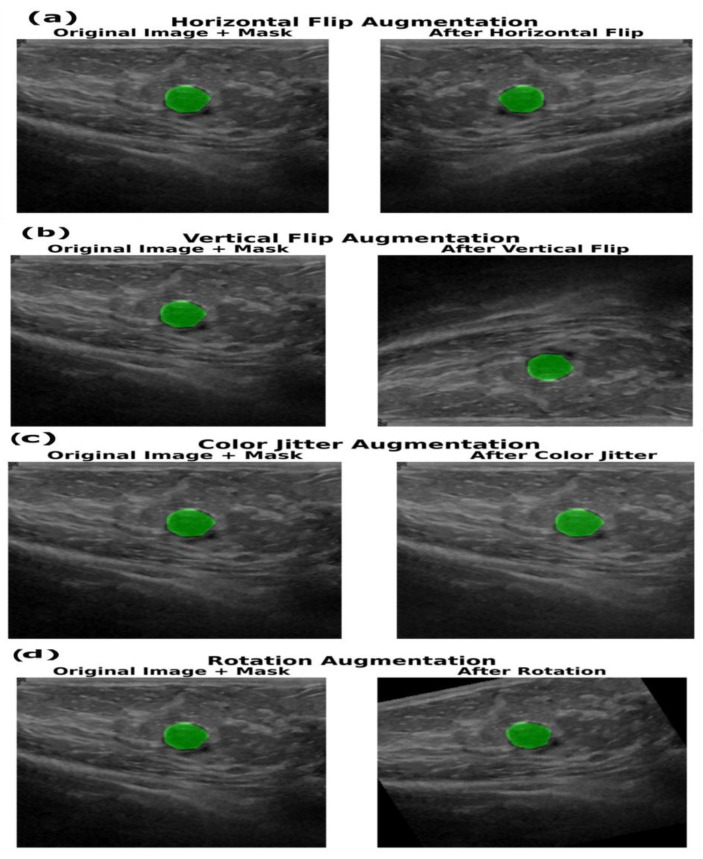
Examples of data augmentation techniques, illustrating common geometric and photometric transformations applied to ultrasound images of the training set and its corresponding segmentation mask (green) for increasing dataset robustness. The displayed techniques are (**a**) horizontal flip, (**b**) vertical flip, (**c**) color jitter (a photometric alteration) and (**d**) rotation augmentation (geometric), all of which help deep learning models generalize better across variations in real-world data capture.

For the breast ultrasound segmentation task, all images were resized to a consistent size based on the results of hyperparameter tuning and normalized using channel-wise means of 0.485, 0.456 and 0.406 and standard deviations of 0.229, 0.224 and 0.225 to standardize pixel intensities. During the training phase, in-memory augmentations were applied to increase variability and reduce overfitting. The applied augmentation techniques include horizontal and vertical flips with a probability of 0.4, random brightness and contrast adjustments with a probability of 0.4 and padding where necessary to ensure a minimum height of 32 pixels and minimum width of 256 pixels, using a constant border value of zero. Validation images were only resized and normalized without random augmentations to ensure consistent evaluation. By applying augmentations at each training iteration, the model effectively observed diverse versions of each input image across epochs, improving generalization while maintaining reproducibility. These augmentation techniques are illustrated in Figure 7.

Similarly, for classification tasks, ultrasound images were converted from grayscale to RGB format, as the pretrained models were trained on color images. Pixel intensities were normalized to a mean of 0.5 and a standard deviation of 0.5, scaling values approximately to [−1, 1]. The applied augmentations were defined with probabilities to increase variability and reduce overfitting. The augmentation techniques were random horizontal and vertical flips (50% each), 90° rotations (50%), and brightness and contrast adjustments (50%). Validation and test sets were only resized and normalized to ensure consistent evaluation. Class labels were automatically assigned from file names (benign’ = 0, ‘normal’ = 1, malignant’ = 2), and weighted random sampling was used during training and validation to mitigate class imbalance, ensuring proportional representation of each class in every batch. The images were resized according to the requirements of the specific classification models. For example, during augmentation, images were resized to 224 × 224 for ResNet18 and to 299 × 299 for InceptionV3 to match the input dimensions expected by each model during the classification task. Both segmentation and multiclass classification tasks were performed using the PyTorch (version 2.9.1+cu126) deep learning framework on an NVIDIA RTX 4060 Ti 16 GB external GPU housed in a Node Titan enclosure connected via Thunderbolt USB-C cable to a Dell Precision 7760 laptop with an 11th Gen Intel Core i7 processor and 32 GB RAM running 64-bit Windows 11.

In the breast cancer image segmentation task, U-Net, U-Net++ and DeepLabV3 decoders were chosen due to their high performance on image segmentation tasks. The decoder architecture was tested with various encoder backbones to assess the impact of different encoder–decoder architectures on breast cancer image segmentation quality and accuracy. The tested encoders include ResNet18, EfficientNet-B0, MobileNetV2 and VGG16. These encoders were selected for the study due to the GPU resource constraints. Larger models such as ResNet50, DenseNet161, etc., were unable to run on the utilized GPU. Moreover, our attempt to train VGG16:DeepLabV3 failed, plausibly due to total memory (VGG16:DeepLabV3) exceeding the 16 GB limit of the GPU used in this study. Hyperparameter optimization was conducted to identify the optimal encoder–decoder architecture. For each decoder, hyperparameters such as the encoder (ResNet18, EfficinetNet-B0, MobileNetV2 or VGG16), learning rate and weight decay (within a range of 0.00001 to 0.001), and batch size (equal to 4, 8, or 16) were optimized to determine the optimal configuration for a GPU setup with 16 GB memory. A batch size of 32 was also attempted but failed due to an out-of-memory error. The optimizer (Adam, AdamW, RMSprop, SGD or Adagrad) and image sizes (288, 320 or 256) were investigated using the OPTUNA optimization framework. The number of epochs was set within an integer range of 10 to 100. The objective of the Optuna study was set to minimize the loss function; a total of 50 Optuna trials were conducted with a trial pruning mechanism to cease dubious trials. The hyperparameter tuning of the segmentation models took between 6 and 14 h, depending on the complexity of the model and the number of hyperparameters. This approach optimizes computational resource allocation by stopping trials that fail to meet predefined improvement criteria for validation loss, accelerating convergence toward the most capable hyperparameter settings. After optimizing the encoder and hyperparameters of each of the decoder architectures, the encoder–decoder models were trained for 2000 epochs, with early stopping set with patience = 25 and the best performing model saved based on the lowest validation loss. Using the best performing segmentation model, the segmentation masks were predicted using training, validation and test datasets. Evaluation metrics including the Jaccard score, area under the curve (AUC) score, IoU, Dice coefficient and pixel accuracy were used to evaluate the segmentation model’s performance on the training, validation and test datasets.

For the multiclass classification of breast ultrasound images, we tested and developed five pretrained models, including ResNet18, InceptionV3, DenseNet121, GoogleNet and MobileNetV3. In the second step of multiclass classification model development, class imbalance is identified and addressed. Class weights were calculated based on the frequency of each class. These weights were used in weighted Random Sampler, a function of PyTorch, to draw samples more frequently from under-represented classes than over-represented classes. This approach helps to balance the dataset by increasing the representation of minority classes during the training process. The next step of the model development process is to initialize the pretrained classifier with respective pretrained weights of the image dataset. For example, DenseNet121 was initialized using the IMAGENET1K_V1 weights pretrained on the ImageNet dataset. The initialized model was tailored for our specific task by replacing the final fully connected layer of the original classifier with a new linear layer to match the number of classes in our dataset. The hyperparameters of each model were optimized using the OPTUNA optimization framework [49]. The search spaces of hyperparameters including the learning rate (1 × 10^−5^ to 1 × 10^−1^), batch size (8,16,32), weight decay (1 × 10^−5^ to 1 × 10^−1^) α of focal loss (0.3 to 0.75), γ of focal loss (3 to 5) and optimizer (SGD, Adam, RMSprop or AdamW) were explored to identify the best combination of hyperparameter values for each model. The objective function of the Optuna study was set to run for 50 trials to minimize the loss function. Each trial of the study was set to run for 100 epochs, with early stopping with a patience value of 10. Based on the setup, early stopping was set to prune a trail if there was no further improvement in the validation loss for 10 consecutive epochs, and it took approximately 8 to 16 h to optimize the hyperparameters of various multiclass classifier models, while the training of segmentation and classification models with the optimized parameters took between 20 and 90 min. However, the inference time on the segmentation task and classification on the external test data (77 samples) took less than 10 min.

After the hyperparameters were optimized using the OPTUNA framework, the optimized model was initialized using the best combination of parameters. In the training phase, focus was placed on maximizing the model’s generalization performance and preventing overfitting through the use of adaptive learning-rate scheduling and early stopping mechanisms. We utilized the ReduceLROnPlateau learning-rate scheduler to reduce the learning rate when the validation loss plateaued. The scheduler was configured with the patience parameter value set to 25. Therefore, the scheduler decreased the learning rate gradually if there was no improvement in the validation loss for 25 epochs. This controlled reduction in the learning rate allowed the model to fine-tune the weights with greater precision and avoid reducing oscillations around local minima and converge. The training loop was set to run for 1000 epochs, with an EarlyStopping mechanism to halt the training when further epochs no longer yielded improvement in the validation loss. EarlyStopping was configured with the patience parameter set to 25, meaning that the training loop broke if there was a further decrease in the validation loss for 25 epochs, thereby preventing overfitting. The EarlyStopping criteria were based on the validation loss (multiclass focal loss), and the best model state was saved at checkpoints, ensuring that best performing model configuration was retained for inference or the testing phase. In the concluding step of the classification task, each model’s performance was analyzed using precision, recall, F1 score and accuracy metrics on the training, testing and validation datasets to understand classification effectiveness and generalizability.

## 4. Results and Discussion

### 4.1. Image Segmentation

We tested the ResNet18, EfficientNet-b0, MobileNetV2 and VGG16 encoders in combination with the U-Net, U-Net++ and DeepLabV3 decoders. Table 3 provides the optimized encoder—decoder combinations and the optimized hyperparameters for the combinations. During Optuna hyperparameter optimization for the U-Net decoder, we determined that the objective value of the study was minimized when the encoder was ResNet18. MobileNetV2 showed the lowest performance during the optimization of the U-Net architecture, with a high objective value of 0.65. For the U-Net++ architecture, the best encoder was ResNet18, with lowest objective value of the Optuna study. The highest objective value for U-Net++ was recorded with the EfficientNet-Bo encoder, indicating the worst performance. Similarly, with DeepLabV3, MobileNetV2 and EfficientNetB0 showed high objective values throughout the optimization study.

**Figure 8:**
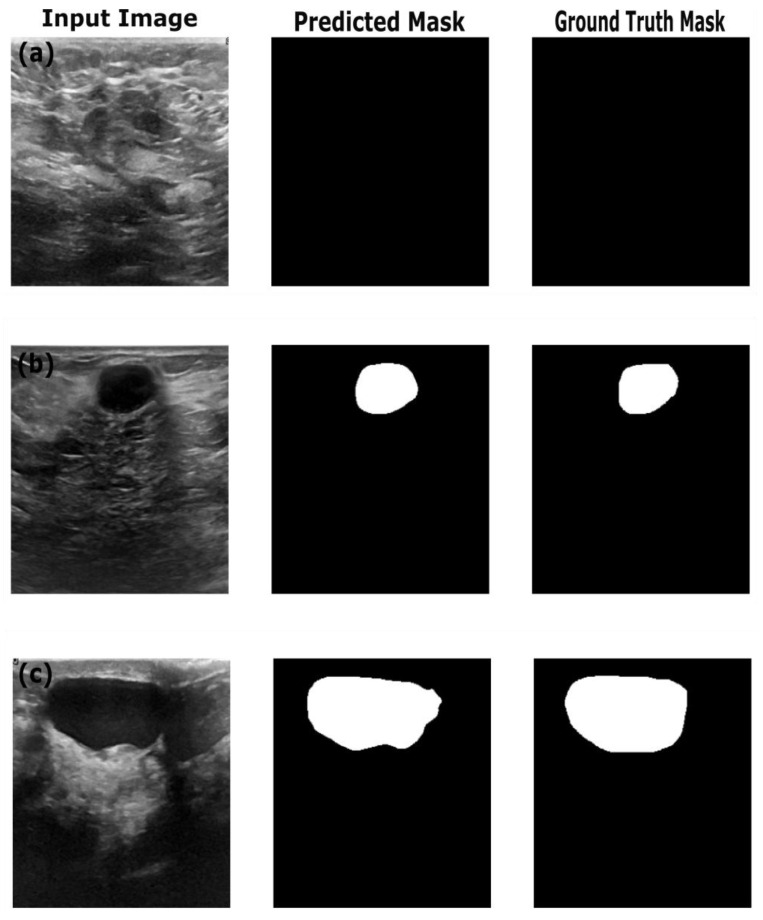
Example predictions of the ground-truth mask by the Resent 18:U-Net model on the (**a**) train dataset, (**b**) validation dataset and (**c**) test dataset.

Table 4 shows the performance of the optimized image segmentation models on the training, validation and test datasets. The ResNet18:U-Net combination achieved a high pixel accuracy of 0.97 on the training set, with a Dice coefficient of 0.86 and an IoU of 0.76, indicating that this model performed well in terms of capturing the relevant pixels and achieved good performance on the training images and masks. However, the Resenet18: U-Net model showed overfitting to the training dataset, with decreased performance on the validation and test datasets. The Dice coefficient dropped to 0.76 and 0.74, and the IoU to dropped 0.62 and 0.59 on the validation and test datasets, respectively. This poor performance on the validation and test data indicates that the model struggled to generalize on the unseen dataset. This trend is reflected in the AUC score—decreasing from 0.91 on training data to 0.85 and 082 on the validation and test datasets. By observing the sample segmentation results (Figure 8) of the ResNet18:U-Net combination, it can be determined that the model produced reasonably accurate in segmentations of both large and small structures; however, it shows edge irregularities and slight oversegmentation visible in larger structures in images from the test set (Figure 8c). Compared to ResNet18:U-Net, Resnet18:U-Net++ achieved better generalization on the test and validation datasets, with improved Dice coefficient and IoU scores. On the training dataset, ResNet18:U-Net++ achieved better performance, with a high pixel accuracy of 0.98, Dice coefficient of 0.87 and IoU of 0.77, similar to ResNet18:U-Net’s performance. However, ResNet18:U-Net++ showed a relatively higher Dice coefficient of 0.83, IoU of 0.71 and AUC score of 0.91, suggesting the Resnet18:U-Net++ architecture mitigates overfitting better than ResNet18:U-Net, especially when applied to an unseen dataset. In comparison with results reported by Almajalid et al. [50], who achieved an average Dice score of 82.5% using a U-Net-based segmentation framework on the BUSI dataset of 221 images, our ResNet18: UNet++ model achieved a Dice coefficient of 83%. The abovementioned authors highlighted U-Net’s robustness and adaptability to ultrasound image segmentation, supporting our findings. Similarly, our results obtained with U-Net are similar to the results reported by Byra et al., who reported a Dice score of 0.77 on the validation dataset [51], while our U-Net architecture achieved a Dice score of 0.76 on the validation dataset. However, our results highlight the importance of advanced architectural variations and advanced models such as ReseNet18:DeepLabV3.

**Figure 9:**
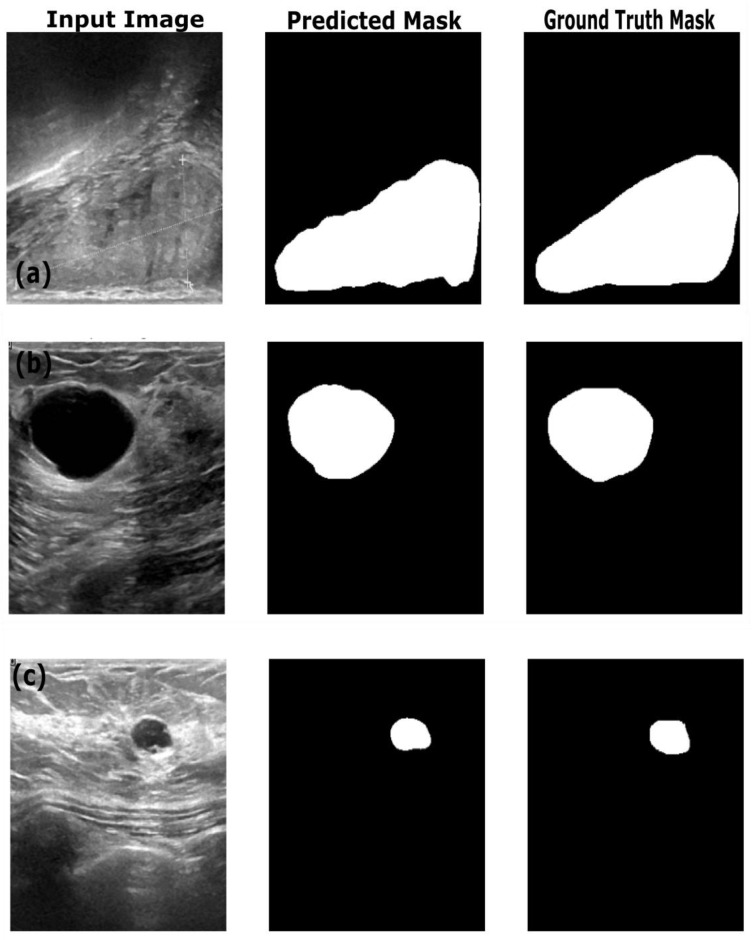
Example predictions of the ground-truth mask by the Resent 18: U-Net++ model on the (**a**) training dataset, (**b**) validation dataset and (**c**) test dataset.

**Figure 10:**
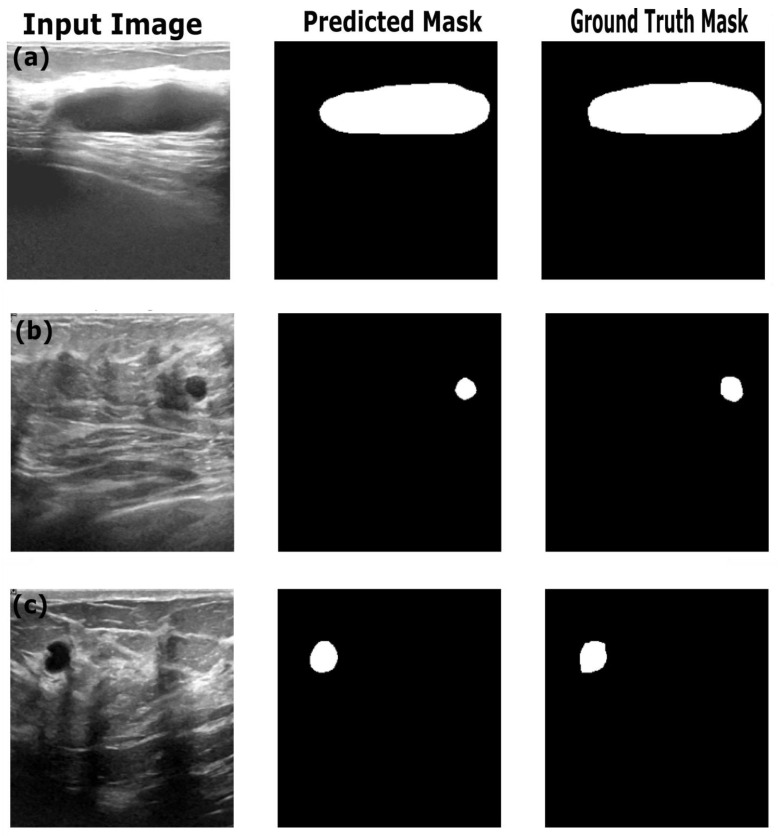
Example predictions of the ground-truth mask by the Resent 18:DeepLabV3 model on the (**a**) training dataset, (**b**) validation dataset and (**c**) test dataset.

**Figure 11:**
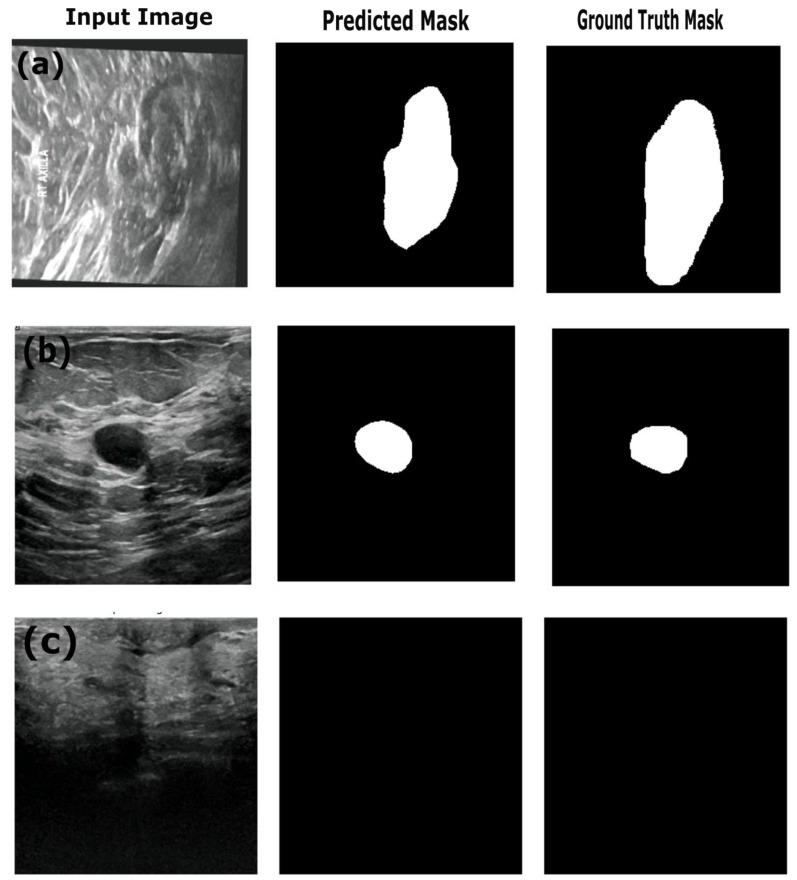
Example predictions of the ground-truth mask by the Resent 18: FADeepLabV3 model on the (**a**) training dataset, (**b**) validation dataset and (**c**) test dataset.

Figure 9 provides the sample results of the ResNet18:U-Net++ architecture on the training (a), validation (b) and test datasets (c), and Figure 9 illustrates that the ResNet18:U-Net++ model accurately segments across different sizes and shapes. The ResNet18:DeepLabV3 model yielded the highest performance overall, with pixel accuracy of 0.98 on both the training and test datasets. The Dice coefficients on the training, validation and test datasets were 0.87, 0.80 and 0.83, respectively. The ResNet18:DeepLabV3 model outperformed ResNet18:U-Net++ in terms of Dice coefficients and IoU on the training dataset. The ResNet18:DeepLabV3 model also maintained high accuracy on test data (examples in Figure 10), and it achieved the highest Dice coefficient and IoU, indicating that ResNet18:DeepLabV3 generalizes better on validation data than other developed models. Our ResNet18:DeepLabV3 results align with results reported in the literature; Badawy et al. [52] reported a mean IoU value of 0.49 before applying fuzzy logic preprocessing [52], whereas our best segmentation model (ReseNet18:DeepLabV3) showed a mean IoU of 0.7. Similarly, they reported that their developed U-Net model achieved a mean IoU of 0.49, while the U-Net model we developed achieved a mean IoU of 0.65. From Figure 10, it is evident that DeepLabV3 strikes a balance between robustness and precision in segmenting large, irregularly shaped lesions, as well as medium and small lesions, with high accuracy and detailed delineation of the edges. The proposed Resnet18:FADeepLabV3 model showed a distinct performance that highlights benefits of the integration of an FEM into DeepLabV3. Our proposed model achieved a Dice score of 0.84 and IoU of 0.71 during training, which is slightly below the baseline Resnet18:DeepLabV3 model. However, it significantly outperformed on AUC, reaching 0.98, compared to the baseline’s 0.93. This indicates our model learned better pixel-level discrimination despite modest reduction in spatial overlap metrics. On the validation data, FADeepLabV3 achieved a Dice score of 0.75 and am IoU of 0.65. However, the AUC on the validation dataset was 0.97, while the baseline achieved 0.87. This shows our model was able to distinguish lesion tissues from the background across different scenarios, even when precise boundary detection remained difficult. The superior performance of the Resnet18:FADeepLabV3 architecture was confirmed during inference on an external test dataset, where it achieved the highest segmentation accuracy across all models (DSC: 0.85; IoU: 0.72). This represents a measurable improvement over the baseline DeepLabV3 (DSC: 0.83; IoU: 0.70), demonstrating that frequency-aware enhancements improve generalization and boundary delineation on unseen ultrasound data. A key result was the model’s test AUC of 0.98, substantially outperforming all competitors, including U-Net++ (AUC: 0.91). This indicates that FADeepLabV3 not only segments more accurately but also produces more reliable confidence estimates, which is critical for interpretation of heterogeneous lesions in clinical ultrasound images. The sample predictions are illustrated in Figure 11.

### 4.2. Image Classification

Table 5 presents results of the OPTUNA optimization framework obtained for the ResNet18, InceptionV3, DenseNet121, GoogleNet and MobilenetV3 models on a multiclass classification task. The F1 macro score was used to assess the models during optimization process.

**Figure 12:**
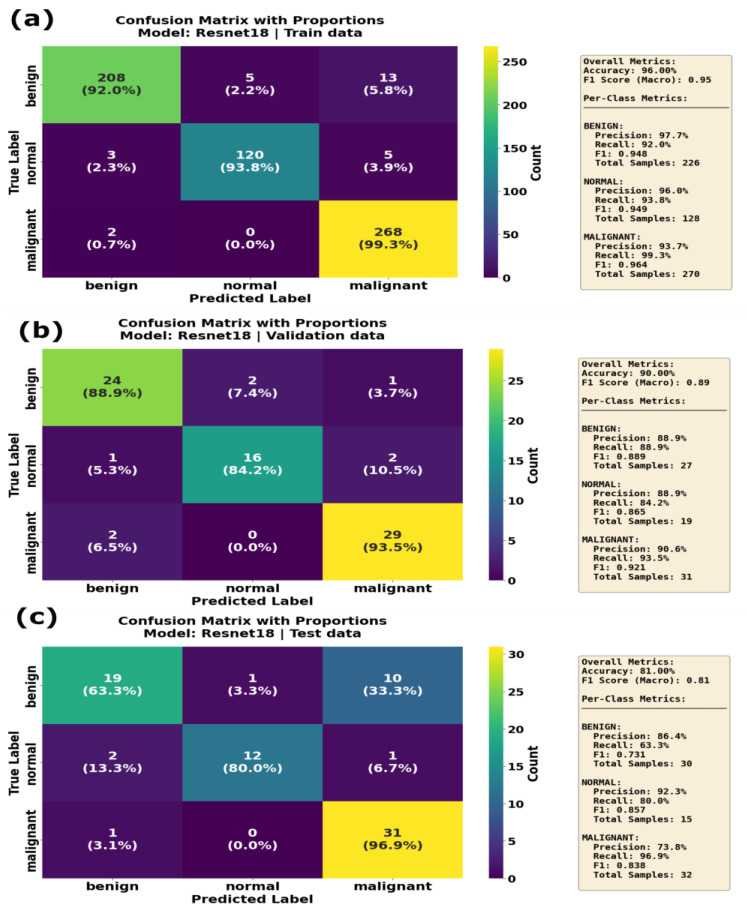
Confusion matrix with proportions for the ResNet18 model on (**a**) training data, (**b**) validation data and (**c**) test data. Each matrix compares the true label (actual class) against the predicted label (model output) for the three classes: benign, normal and malignant. Overall accuracy of 96% on the training set, which dropped to 88.9% on the validation set and 86.4% on the test set. Specifically for the malignant class, the model demonstrated high recall across all subsets, maintaining 93.8% on training data, 93.5% on the validation set and 96.9% on the critical test set.

ResNet18 achieved the lowest F1 score of 0.77 during the optimization process utilizing the RMSprop optimizer in combination with a learning rate of 0.0000491, weight decay of 0.00027 and training batch size of 32. GoogleNet, optimized with a Adam optimizer, a learning rate of 0.000163, weight decay of 0.00009, focal loss parameters (α and γ) equal to 0.73 and 4.24, β_1_ = 0.91 and β_2_ = 0.93, achieved a slightly better F1 score of 0.82. On the other hand, InceptionV3 achieved performance similar to that of GoogleNet, with only a one-point increase in the F1 score. During the optimization process, MobileNetV3 showed the best F1 score of 0.88 and outperformed other models. MobileNetV3 was optimized with RMSProp; a learning rate of 0.0000246; weight decay of 0.00060; FL (α) = 0.44; FL (γ) = 4.86; and β_1_ and β_2_ values equal to 0.81 and 0.96, respectively. The effective performance of the AdamW optimizer for MobileNetV3 highlights the model’s compatibility with robust weight decay, contributing to generalization while minimizing overfitting. Optuna optimization results revealed that optimal hyperparameters vary significantly by model architecture, and MobileNetV3 achieved the highest accuracy with the lowest batch size of 8 on both the training and validation datasets. ResNet18 achieved better performance on the training dataset, with an F1 score of 0.95, accuracy of 0.90 and well-balanced precision and recall across all classes for the training dataset. ResNet18’s validation performance was also high, with an F1 score of 0.89 and accuracy of 0.82. However, the model’s performance on the test dataset dropped, with F1 score = 0.81 and accuracy = 0.77. Moreover, the recall on the benign class was 0.63, indicating that while ResNet18 generalized well, it had difficulty recognizing benign classes in the test dataset. The confusion matrix results (Figure 12) of ResNet18 show that the model correctly identified 208 benign, 120 normal and 268 malignant samples, with fewer misclassifications during the training phase. ResNet18 misclassified only two malignant cases in the training and validation datasets. However, during the inference phase, ReseNet18 misclassified 10 benign cases as malignant, even though it showed better performance for normal and malignant classes. Badawy et al. developed ResNet101; in comparison, for the lightweight model we developed in this study, we chose ReseNet18 due to memory constraints of the GPU. Badawy et al. reported that ResNet101 achieved better performance the other ResNet variants they tested (ResNet18 and ResNet50) on the BUSI dataset [52].

**Figure 13:**
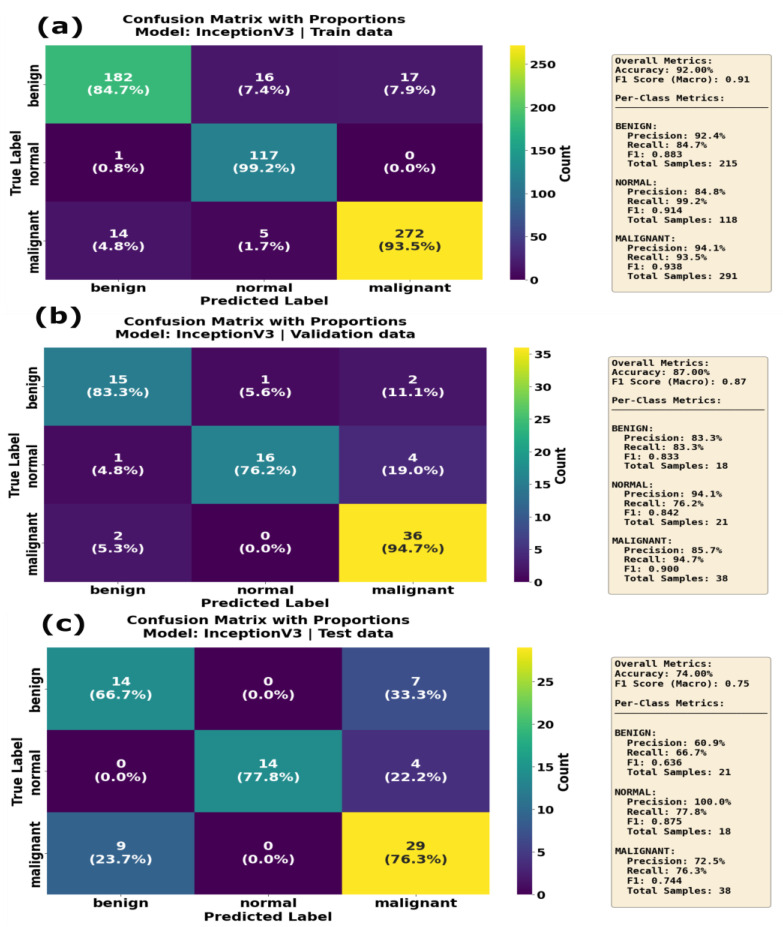
Confusion matrix with proportions for the InceptionV3 model on (**a**) training data, (**b**) validation data and (**c**) test data. Each matrix compares the true label (actual class) against the predicted label (model output) for the three classes: benign, normal and malignant. Overall accuracy reached 92.08% on the training set, decreased to 87.80% on the validation set and further declined to 74.00% on the test set. The malignant class demonstrated strong recall of 93.5% on training data but degraded to 94.7% on validation data and 76.3% on the test set, indicating overfitting tendencies.

**Figure 14:**
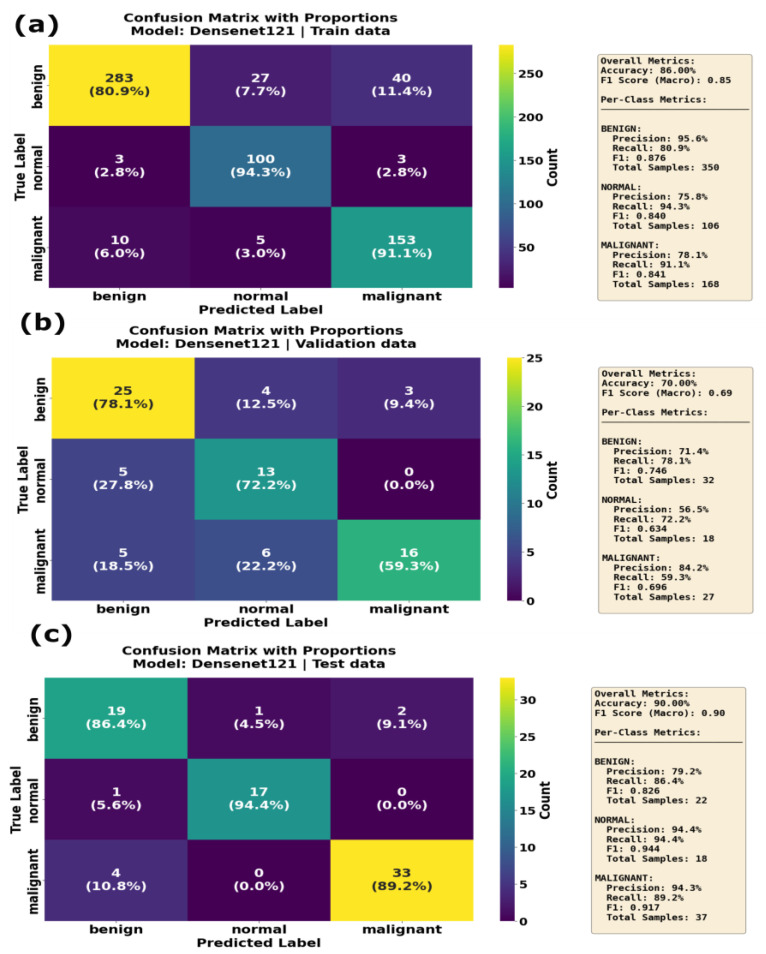
Confusion matrix with proportions for the DenseNet121 model on (**a**) training data, (**b**) validation data and (**c**) test data. The model achieved 86.80% overall accuracy on the training set, declining to 70.00% on validation data and improving to 90.00% on the test set. The malignant class showed 91.1% recall on training data, declining to 59.3% on validation data but recovering to 89.2% on the test set, demonstrating variable performance across datasets.

The InceptionV3 model achieved an F1 score of 0.91 and an accuracy of 0.91 on the training data, with a high recall in the normal class (0.99; see Table 6). On the validation dataset, InceptionV3 showed an F1 score of 0.86, with a validation accuracy of 0.74, indicating a moderate overfitting problem. During the inference phase, InceptionV3 showed lower performance, with a F1 score and accuracy of 0.75 and 0.83, respectively. InceptionV3 performed well on the normal class label, with precision = 1.0, but the recall for the benign class was equal to 0.63, indicating that InceptionV3 struggled to differentiate the benign class during the inference phase. As shown by the confusion matrix in Figure 13, the InceptionV3 model correctly identified 182 benign, 117 normal and 272 malignant cases, with minimal misclassification on training dataset. The validation data confusion matrix for the InceptionV3 model illustrates that the model correctly classified 36 out of 38 malignant cases. However, during the inference phase, it faced challenges in classifying the benign class, with only 14 of the benign cases classified correctly, with 7 cases where it misclassified benign samples as malignant samples. DenseNet121 performed reasonably well on the training and test (unseen) datasets. On the training dataset, the DenseNet121 model achieved an F1 score and accuracy of 0.86 and 0.85, respectively. On the validation dataset, it achieved an F1 score of 0.73 and an accuracy of 0.71. However, the model demonstrated strong performance on the test data when compared to validation data, indicating that the model generalizes well when applied to unseen dataset. Examining the confusion matrix (Figure 14a) of the DenseNet121 model on the training dataset, it is evident that DenseNet121 correctly identified 283 benign, 100 normal and 153 malignant samples, although it misclassified 27 benign images as normal and 40 benign samples as malignant. On the validation dataset (Figure 14b), DenseNet121 showed mixed performance, with 25 benign images correctly classified, and the model misclassified 5 benign samples as normal and 6 malignant samples as normal. During the inference phase, the DenseNet121 model achieved better performance, with only a few misclassified samples. It correctly classified 19 benign, 17 normal and 33 malignant samples, implying a high level of generalization.

**Figure 15:**
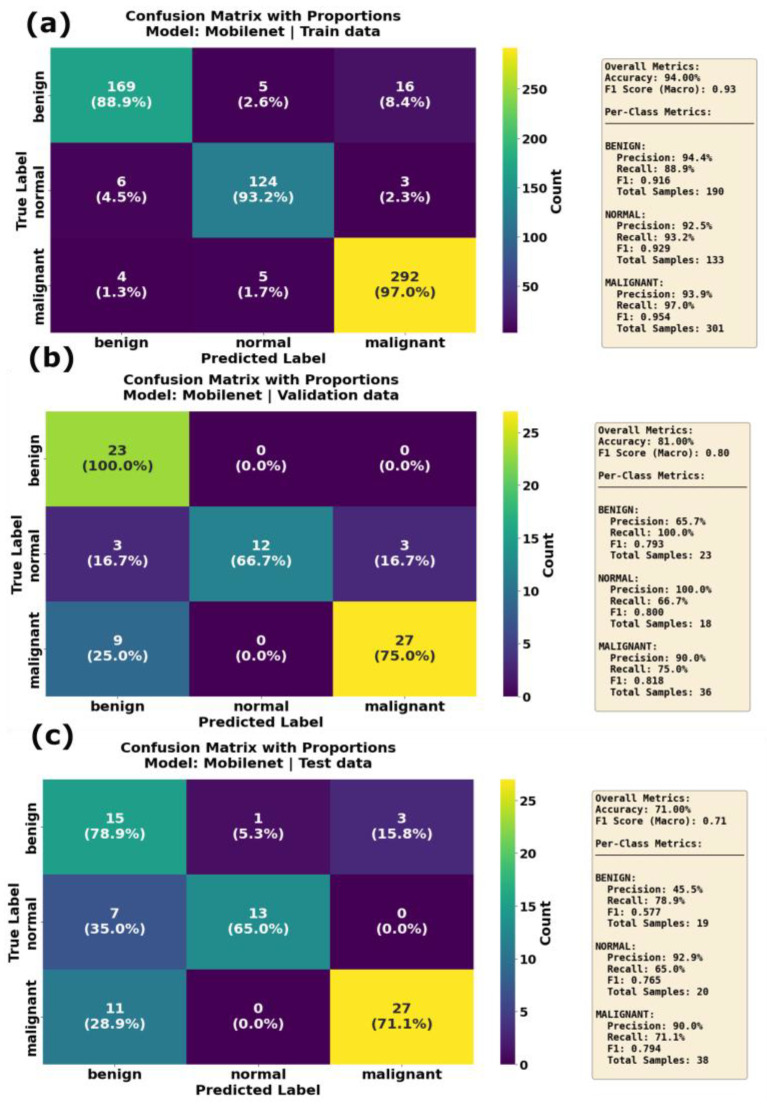
Confusion matrix with proportions for the MobileNet model on (**a**) training data, (**b**) validation data and (**c**) test data. Overall accuracy reached 94.08% on training data, dropped significantly to 81.00% on validation data and further declined to 71.00% on the test set. The malignant class achieved an exceptional 97.0% recall on training data but degraded to 75.0% on validation data and 71.1% on the test set, indicating substantial performance degradation on unseen data.

The MobileNetV3 model presented consistent performance across all classes, with precision and recall above 0.9 for the training dataset. The accuracy and F1 score on the training dataset were 0.93 and 0.94, respectively. However, the MobileNetV3 model showed poor performance on the test dataset, with a lower F1 score and accuracy due to the impact of lower precision for the benign class. This indicates that the MobilenetV3 model struggled to classify the benign class on the unseen dataset. The results of the developed MobileNetV3 model align with other reports in the literature. Yan et al. reported that MobileNetV1 achieved better classification performance than MobileNetV2 [53]. From Figure 15a, it is evident that the developed MobileNetV3 model correctly identified 168 benign, 129 normal and 292 malignant cases. It showed strong performance on validation data (Figure 15b) for benign and malignant classes. However, in the inference phase, the MobileNetV3 model misclassified 1 benign case as normal, 7 normal cases as benign and 11 normal cases as malignant, showing confusion primarily in distinguishing the normal class.

**Figure 16:**
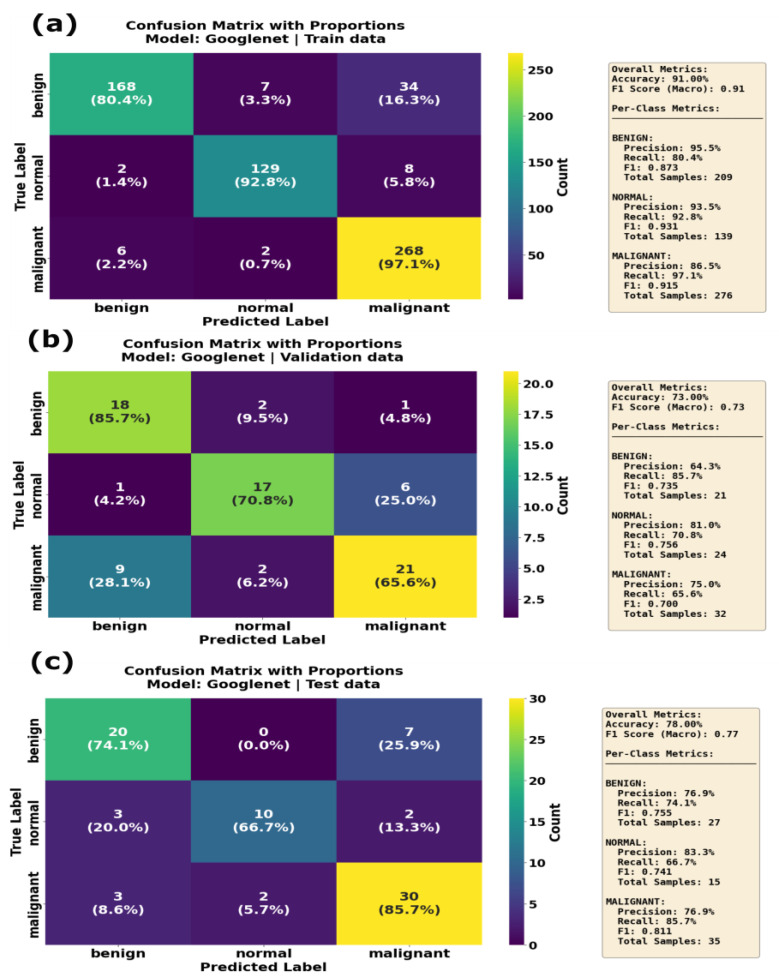
Confusion matrix with proportions for GoogleNet model on (**a**) training data, (**b**) validation data and (**c**) test data. Overall accuracy was 91.00% on training data, decreased to 73.00% on validation data and reached 78.00% on the test set. The malignant class maintained a strong 97.1% recall on training data but dropped to 65.6% on validation data and improved to 85.7% on the test set, showing inconsistent generalization performance across datasets.

The GoogleNet model, also known as the InceptionV1model, showed a high training F1 and accuracy scores of 0.91 and 0.91, respectively. However, the recall for benign cases was relatively low, at 0.8, implying that the model struggled to detect benign features during the training phase. The F1 score and accuracy of the GoogleNet model on the validation dataset were 0.73 and 0.73, respectively. During the inference phase, GoogleNet achieved an F1 score of 0.77 and an accuracy of 0.77, with balanced precision across all the classes but lower recall for normal and malignant classes. Thus, the GoogleNet model was unable to differentiate the classes during the inference phased. Similarly, the confusion matrix results of the GoogleNet model on training dataset evidence the fact that model showed balanced performance across all the classes in the training phase (Figure 16a), correctly classifying 168 benign, 129 normal and 268 malignant cases, with minimal misclassification. On the validation dataset, GoogleNet correctly classified 18 benign, 17 normal and 21 malignant samples; however, some of the malignant samples were misclassified as normal (Figure 16b). During the inference phase, the GoogleNet model’s performance was slightly reduced, with 20 benign, 10 normal and 30 malignant cases correctly identified. However, it misclassified seven benign cases as malignant, three normal cases as benign and two malignant classes as normal. Figure 11 shows a comparison of all the developed models’ performance on the training, validation and test datasets.

**Figure 17:**
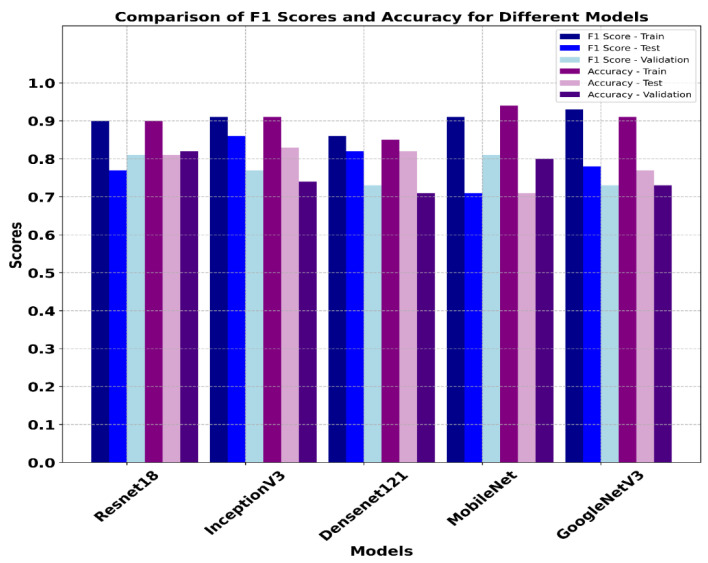
Comparison of models’ performance on training, validation and test datasets based on F1 score and accuracy.

**Figure 18:**
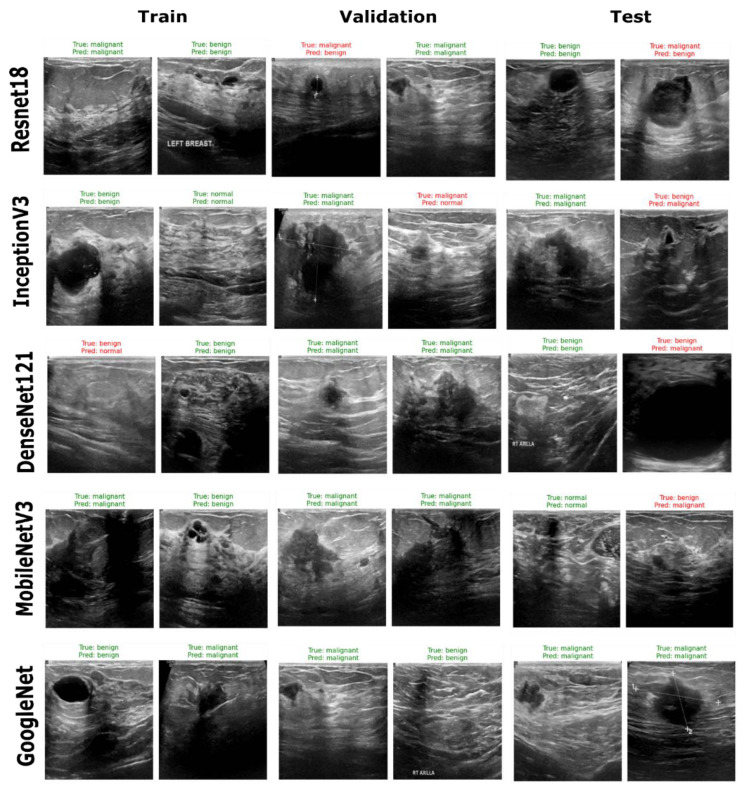
Samples of classification performance of five encoder architectures (ResNet18, InceptionV3, DenseNet121, MobileNetV3 and GoogleNet) across training, validation and test datasets. Green labels indicate correct predictions, while red labels denote misclassifications. The figure demonstrates each model’s ability to discriminate between normal, benign and malignant breast lesions in ultrasound images.

Figure 17 illustrates that the ResNet18 and GoogleNetV3 models achieved the highest F1 scores on training data, with GoogleNetV3 achieving a slightly higher F1 score of 0.93 and an accuracy of 0.91. However, the ResNet18 model demonstrated better generalization on the unseen dataset, attaining validation and test accuracies of 0.82 and 0.81, respectively. InceptionV3 also performed well, while DenseNet121 displayed moderate scores on validation and test data, with F1 scores of 0.73 and 0.82, respectively. Similarly, the MobileNetV3 model, despite a better training accuracy of 0.94, did not show robust performance on the test data, with a lower accuracy of 0.71. Considering the accuracies and F1 scores, the ResNet18 model can be selected as the most balanced model, with stable performance across training, validation and test datasets. The model’s consistency in accuracy and F1 scores across three datasets supports its reliability for the multiclass classification of breast cancer ultrasound imagery data. Sample predictions of the developed classification models are provided in Figure 18.

#### Misclassification Analysis

**Figure 19:**
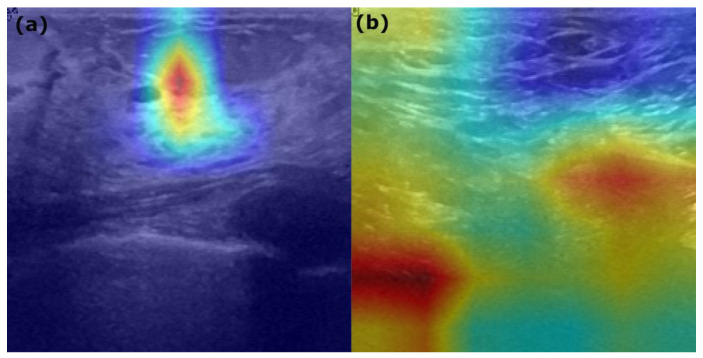
Grad-CAM visualizations of model misclassifications. (**a**) Benign-to-normal: localized focal attention (yellow–orange hot spot) indicates underestimation of pathological significance. (**b**) Benign-to-malignant: diffuse dual hot spots reflect overinterpretation of lesion boundary features as malignancy indicators.

As shown in Table 7, three benign cases were misclassified as normal with high confidence (0.75 normal probability). The model assigned minimal benign probability (0.16) and very low malignant probability (0.09), indicating complete dismissal of benign characteristics. These lesions likely presented with subtle morphological features that the network failed to recognize as pathological, treating them as indistinguishable from normal tissue. Two malignant cases were predicted as normal, with probabilities of 0.10 and 0.10, respectively. However, the model assigned moderate malignant probabilities (0.51), suggesting partial recognition of malignant features but ultimately favoring the normal classification. This represents a critical clinical misdiagnosis, where dangerous lesions were underestimated. One normal image was predicted as malignant with 0.46 probability, with benign probability at 0.38. The model showed borderline confidence, with significant probability mass across multiple classes, indicating poor discriminative ability in distinguishing normal tissue from pathology. Two normal cases were predicted as benign. The first showed a moderate benign probability (0.42), while the second showed higher uncertainty with more balanced probabilities (0.37 benign and 0.34 malignant). Both represent false-positive benign predictions, suggesting oversensitivity to minor textural variations or artifacts. Figure 19a shows a Grad-CAM visualization (benign as normal misclassification), displaying a concentrated yellow–orange focal hot spot surrounded by cooler blue regions, indicating the model’s attention was narrowly focused on a specific hypoechoic area. This localized activation pattern suggests the classifier recognized a discrete lesion but underestimated its clinical significance.

Figure 19b shows a Grad-CAM visualization of benign-to-malignant misclassification, exhibiting dual red hot spots with broader diffuse warm-colored activation across the field, reflecting the network’s emphasis on lesion boundary characteristics and heterogeneous internal echoes, leading to overinterpretation of borderline morphological features as malignancy indicators.

## 5. Conclusions and Future Work

With the aim of assisting healthcare practitioners and radiologists in early detection of breast cancer, we conducted an extensive study on the application of transfer learning techniques based on the pretrained models to accurately conduct breast cancer segmentation and classification of breast cancer ultrasound images. We focused on data augmentation, hyperparameter optimization with Optuna and the construction of a robust training process to address challenges presented by the limited size and class imbalance of the BUSI dataset. For the image segmentation task, the DeepLabV3 model outperformed the U-Net and U-Net++ models by achieving high pixel accuracy and good generalization on training, validation and test datasets. U-Net predictions showed edge irregularities and oversegmentation, while the ResNet18:DeepLabV3 model showed strong performance in terms of delineating both large and small lesions with minimal edge irregularities, indicating the model’s suitability for breast cancer image segmentation. The DeepLabV3 ResNet18 model achieved a Dice coefficient of 0.83, IoU of 0.71 and pixel accuracy of 0.98 on the test dataset, demonstrating its ability to generalize well on the unseen dataset. The proposed ResNet18–FADeepLabV3 model further enhanced segmentation performance by integrating frequency-domain feature processing into the DeepLabV3 decoder. Although its training Dice coefficient and IoU were slightly lower than those of the baseline DeepLabV3 model, FADeepLabV3 achieved superior generalization on the test set, with a Dice coefficient of 0.85 and an IoU of 0.72. Notably, the model attained an AUC of 0.98, substantially outperforming all other models and indicating highly reliable discrimination between lesion and non-lesion regions. These results highlight the strength of frequency-aware feature enhancement in addressing ultrasound-specific challenges such as speckle noise and low contrast. For the classification of breast cancer images, the ResNet18 model achieved an F1 score of 0.81 and an overall pixel accuracy of 0.77 on the test dataset, and ResNet18 showed better generalization on the unseen dataset when compared to MobileNetV3 and GoogleNet. The MobileNetV3 model achieved the highest F1 score of 0.94 on the training data but showed low accuracy (0.71) on the test data. Future work will focus on integrating attention mechanisms into the encoder and decoder of FADeepLabV3. We also plan to explore adaptive frequency methods that allow models to automatically learn which features are most useful. In addition, more extensive hyperparameter tuning will be performed to further improve FADeepLabV3’s stability and accuracy.

## Figures and Tables

**Table 1:** Data distribution of image-mask pairs for binary segmentation task.

Training Dataset		Validation Dataset		Test Dataset	
Images	Masks	Images	Masks	Images	Masks
624	624	77	77	77	77

**Table 2:** Dataset distribution of the images for multiclass classification task.

Ultrasound Breast Images	Training Dataset	Validation Dataset	Test Dataset
Benign	350	43	43
Malignant	168	21	21
Normal	106	13	13
Total	624	77	77

**Table 3:** Optuna hyperparameter optimization of segmentation models.

Encoder	Decoder	Learning Rate	Weight Decay	Batch Size	Optimizer	Image Size
Resnet18	U-Net	0.000081	0.000079	8	Adam	288
Resnet18	U-Net++	0.000155	1.09011	8	RMSprop	288
Resnet18	DeepLabV3	0.00019	0.00019	8	AdamW	256

**Table 4:** Performance of segmentation models on training, validation and test datasets.

Encoder–Decoder	Dataset	Pixel Accuracy	Dice Coefficient	IoU	AUC Score
Resnet18:U-Net	Train	0.97	0.86	0.76	0.91
	Validation	0.96	0.76	0.62	0.85
	Test	0.96	0.74	0.59	0.82
Resnet18:U-Net++	Train	0.98	0.87	0.77	0.92
	Validation	0.96	0.76	0.60	0.84
	Test	0.97	0.83	0.71	0.91
Resnet18:DeepLabV3	Train	0.98	0.87	0.78	0.93
	Validation	0.97	0.80	0.67	0.87
	Test	0.98	0.83	0.70	0.90
Resnet18:FADeepLabv3	Train	0.97	0.84	0.71	0.98
	Validation	0.96	0.75	0.65	0.97
	Test	0.97	0.85	0.72	0.98

**Table 5:** Optuna hyperparameter optimization of multiclass breast cancer classification models.

Model	Optimizer	Learning Rate	Weight Decay	FL (α) *	FL (γ) *	Batch Size	$\beta_{1}$	$\beta_{2}$	F1 Score
Resnet18	RMSprop	0.0000491	0.00027	0.57	4.03	32	N/A	N/A	0.77
InceptionV3	Adam	0.0000163	0.00009	0.33	4.41	16	0.84	0.98	0.83
Densenet121	RMSprop	0.0000246	0.00060	0.65	3.55	32	N/A	N/A	0.87
MobilenetV3	AdamW	0.0001120	0.00626	0.44	4.86	8	0.81	0.96	0.88
GoogleNet	Adam	0.0008705	0.00015	0.73	4.24	32	0.91	0.93	0.82

* FL (α) is the weighting factor that balances different classes. When the dataset is imbalanced, FL (γ) is the parameter that focuses on the instances that are hard to classify. $\beta_{1}$ is first moment decay rate, whereas $\beta_{2}$ is the RMSprop decay rate.

**Table 6:** Performance of the breast cancer classifiers on the training, validation and test datasets.

Model	Dataset	F1 Score	Accuracy	Metric	Benign	Normal	Malignant	Macro AVG
Resnet18	Train	0.90	0.90	Precision	0.98	0.96	0.94	0.96
				Recall	0.92	0.94	0.99	0.95
				F1 score	0.95	0.95	0.95	0.95
	Test	0.77	0.81	Precision	0.86	0.92	0.74	0.84
				Recall	0.63	0.80	0.97	0.80
				F1 score	0.73	0.86	0.84	0.81
	Validation	0.81	0.82	Precision	0.89	0.89	0.91	0.89
				Recall	0.89	0.84	0.94	0.89
				F1 score	0.89	0.86	0.92	0.89
InceptionV3	Train	0.91	0.91	Precision	0.92	0.85	0.94	0.90
				Recall	0.85	0.99	0.93	0.92
				F1 score	0.88	0.91	0.94	0.91
	Test	0.86	0.83	Precision	0.61	1.0	0.72	0.78
				Recall	0.67	0.78	0.76	0.74
				F1 score	0.64	0.88	0.74	0.75
	Validation	0.77	0.74	Precision	0.83	0.94	0.86	0.88
				Recall	0.83	0.76	0.95	0.85
				F1 score	0.83	0.84	0.90	0.86
Densenet121	Train	0.86	0.85	Precision	0.96	0.76	0.78	0.83
				Recall	0.81	0.94	0.91	0.89
				F1 score	0.88	0.84	0.84	0.85
	Test	0.82	0.82	Precision	0.79	0.94	0.94	0.89
				Recall	0.86	0.94	0.89	0.90
				F1 score	0.83	0.94	0.92	0.90
	Validation	0.73	0.71	Precision	0.71	0.57	0.84	0.71
				Recall	0.78	0.72	0.59	0.70
				F1 score	0.75	0.63	0.70	0.69
MobileNetV3	Train	0.93	0.94	Precision	0.94	0.93	0.94	0.94
				Recall	0.89	0.93	0.97	0.93
				F1 score	0.92	0.93	0.95	0.93
	Test	0.71	0.71	Precision	0.45	0.93	0.90	0.76
				Recall	0.79	0.65	0.71	0.72
				F1 score	0.58	0.76	0.79	0.71
	Validation	0.81	0.80	Precision	0.66	1.00	0.90	0.85
				Recall	1.00	0.67	0.75	0.81
				F1 score	0.79	0.80	0.82	0.80
GoogleNet	Train	0.91	0.91	Precision	0.95	0.93	0.86	0.92
				Recall	0.80	0.93	0.97	0.90
				F1 score	0.87	0.93	0.91	0.91
	Test	0.78	0.77	Precision	0.77	0.83	0.77	0.79
				Recall	0.74	0.67	0.86	0.75
				F1 score	0.75	0.74	0.81	0.77
	Validation	0.73	0.73	Precision	0.64	0.81	0.75	0.73
				Recall	0.86	0.71	0.66	0.74
				F1 score	0.73	0.76	0.70	0.73

**Table 7:** Misclassification analysis of Resent18 on validation dataset.

S.No	True Label	Predicted Label	Normal Probability	Benign Probability	Malignant Probability
1	0	1	0.38	0.42	0.20
2	0	1	0.32	0.44	0.24
3	0	1	0.35	0.38	0.27
4	1	2	0.10	0.39	0.51
5	2	0	0.81	0.02	0.17
6	1	2	0.10	0.39	0.51
7	2	0	0.81	0.02	0.17
8	1	2	0.10	0.39	0.51
9	2	0	0.81	0.02	0.17

## Data Availability

The data presented in this study are openly available in https://www.sciencedirect.com/science/article/pii/S2352340919312181 (accessed on 5 May 2025).
